# Long-acting κ opioid antagonists nor-BNI, GNTI and JDTic: pharmacokinetics in mice and lipophilicity

**DOI:** 10.1186/1471-2210-12-5

**Published:** 2012-05-29

**Authors:** Thomas A Munro, Loren M Berry, Ashlee Van’t Veer, Cécile Béguin, F Ivy Carroll, Zhiyang Zhao, William A Carlezon, Bruce M Cohen

**Affiliations:** 1 McLean Hospital, Belmont, MA & Department of Psychiatry, Harvard Medical School, Boston, MA, USA; 2 Pharmacokinetics and Drug Metabolism, Amgen Inc, Cambridge, MA, USA; 3 Organic and Medicinal Chemistry, Research Triangle Institute, Research Triangle Park, NC, USA

**Keywords:** Norbinaltorphimine, Nor-BNI, 5’-guanidinonaltrindole, 5’-GNTI, JDTic, Pharmacokinetics, Lipophilicity, P-gp, JNK1, MAPK8

## Abstract

**Background:**

Nor-BNI, GNTI and JDTic induce κ opioid antagonism that is delayed by hours and can persist for months. Other effects are transient. It has been proposed that these drugs may be slowly absorbed or distributed, and may dissolve in cell membranes, thus slowing elimination and prolonging their effects. Recent evidence suggests, instead, that they induce prolonged desensitization of the κ opioid receptor.

**Methods:**

To evaluate these hypotheses, we measured relevant physicochemical properties of nor-BNI, GNTI and JDTic, and the timecourse of brain and plasma concentrations in mice after intraperitoneal administration (using LC-MS-MS).

**Results:**

In each case, plasma levels were maximal within 30 min and declined by >80% within four hours, correlating well with previously reported transient effects. A strong negative correlation was observed between plasma levels and the delayed, prolonged timecourse of κ antagonism. Brain levels of nor-BNI and JDTic peaked within 30 min, but while nor-BNI was largely eliminated within hours, JDTic declined gradually over a week. Brain uptake of GNTI was too low to measure accurately, and higher doses proved lethal. None of the drugs were highly lipophilic, showing high water solubility (> 45 mM) and low distribution into octanol (log D_7.4_ < 2). Brain homogenate binding was within the range of many shorter-acting drugs (>7% unbound). JDTic showed P-gp-mediated efflux; nor- BNI and GNTI did not, but their low unbound brain uptake suggests efflux by another mechanism.

**Conclusions:**

The negative plasma concentration-effect relationship we observed is difficult to reconcile with simple competitive antagonism, but is consistent with desensitization. The very slow elimination of JDTic from brain is surprising given that it undergoes active efflux, has modest affinity for homogenate, and has a shorter duration of action than nor-BNI under these conditions. We propose that this persistence may result from entrapment in cellular compartments such as lysosomes.

## Background

A growing body of preclinical evidence suggests that selective κ (kappa) opioid antagonists may have therapeutic potential against conditions such as depression and anxiety disorders [1]. However, the drugs used in this research exhibit an extraordinarily long duration of action, which has complicated experimental design and interpretation. The effects of non-selective opioid antagonists typically persist for only a few hours in vivo; durations of several days are considered extremely long. In striking contrast, the effects of selective κ opioid antagonists can persist for weeks or months [2,3]. A less-noted peculiarity of their timecourse is extremely delayed onset; maximal antagonism can be delayed by hours or days even after central administration, compared to minutes for nonselective antagonists [3]. Recently, short-acting κ opioid antagonists have been reported, which appear in preliminary experiments to exert similar effects on stress-related behaviors [4,5]. However, it is as yet unclear whether the abnormal timecourse of the earlier agents is a desirable feature or a liability for clinical development [1]. Additionally, a consensus has not yet been established on the mechanism of this extremely unusual timecourse.

**Figure 1:**
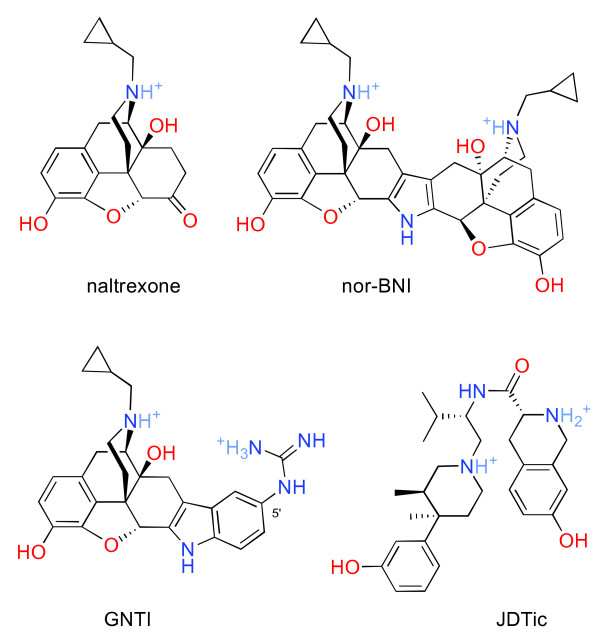
**Structures of nor-BNI, GNTI, JDTic, and naltrexone.** Drugs are shown in the predominant ionization state at physiological pH.

The first reported highly selective κ opioid antagonist was norbinaltorphimine (nor-BNI, Figure 1), a dimeric naltrexone derivative [6]. The compound has become a standard tool in opioid pharmacology, thoroughly characterized in a large body of research. In vitro, nor-BNI reliably produces surmountable antagonism of κ opioids, with high potency and selectivity over μ (mu) and δ (delta) opioid receptors [2]. Of the many other selective κ opioid antagonists which have been developed since [2,7], two have been the subject of considerable in vivo study: 5’-guanidinonaltrindole (GNTI) [8] and JDTic [9], both shown in Figure 1. In vitro, these compounds also produce surmountable antagonism of κ opioids, with sub-nanomolar potency and high selectivity over μ and δ [2].

### Delayed onset

In vivo, nor-BNI can exert selective antagonism of diverse κ opioids across numerous assays and species [3]. In these respects, nor-BNI is unremarkable compared to other selective opioid antagonists. However, the compound’s timecourse is extremely different. Negligible antagonism of κ opioids is evident for 30 minutes after subcutaneous administration; antagonism then rises gradually towards a plateau after at least two hours [10]. By contrast, the effects of naltrexone, a non-selective antagonist, peak within 30 minutes, then decline rapidly [11]. Onset of κ antagonism is markedly delayed even after central administration of nor-BNI. Whereas the effects of naloxone peak within 15 minutes of i.c.v. administration and decline rapidly [12], nor-BNI again shows dramatically slower onset, with κ antagonism increasing for at least 4 hours [13]. This delay in onset has been observed in numerous studies in multiple species [3]. GNTI and JDTic also show robust and selective antagonism of κ opioids in vivo [3]. Like nor-BNI, maximal κ antagonism after systemic administration of GNTI is delayed by several hours [14]; for JDTic, the delay is substantially greater than six hours after oral administration [15].

### Ultra-long duration of action

These three compounds exhibit an extremely long duration of action, producing measurable antagonism for several weeks at minimally-effective doses [2,3]. At higher doses and by other routes of administration, the effects of these compounds are further prolonged. This has been most extensively studied for nor-BNI, which at high doses has a duration of action of several months in some species [3,16]. Compare these values to the typical duration of several hours achieved by the non-selective opioid antagonists naltrexone and naloxone [11]. Indeed, these durations are substantially longer even than that of the irreversible antagonist β-chlornaltrexamine (β-CNA). This drug binds covalently to opioid receptors, and persists until the receptors themselves are degraded and replaced, giving what has been described as an “ultra long” duration of action [17]. At the highest sub-lethal i.c.v. dose, β-CNA exhibited μ antagonism for less than 6 days [17]. This duration is greatly exceeded by even minimally-effective doses of nor-BNI, GNTI and JDTic [18].

### Transient side-effects

Despite its high κ-selectivity in vitro, nor-BNI produces transient μ antagonism in vivo. The timecourse is strikingly different than at κ, peaking approximately 30 minutes after subcutaneous administration and lasting approximately 4 hours [10]. This is approximately the same timecourse as naltrexone [11]. GNTI reportedly does not cause transient μ antagonism [14]; however, it does cause another transient side-effect, sedation. This is of rapid onset and lasts several hours [14]. Transient μ antagonism has not been reported after JDTic [19]; nor are we aware of other transient side-effects. It should be noted, however, that less evidence is available for this compound. That nor-BNI and GNTI induce rapid-onset, transient side-effects along with delayed and prolonged κ antagonism is a notable and puzzling characteristic which has not yet been explained.

### Proposed mechanisms

Several mechanisms for the extremely unusual timecourse of these compounds have been proposed, none of which have won broad acceptance. They can be divided into two general categories. In the first category, the timecourse of antagonism is presumed to reflect the timecourse of the drugs themselves at the effect site. It has been proposed that the delay in onset of nor-BNI may be due to the large size of the molecule (compare naltrexone, Figure 1), or poor membrane permeability, causing slow diffusion to the site of action [20]. The long duration of action has been tentatively attributed to dissolution in cell membranes, creating a depot from which the drug would slowly diffuse [13].

The second general category involves irreversible processes: that the drugs, while transient themselves, initiate some process which produces delayed and persistent antagonism. It was speculated that these drugs might induce an abnormal conformation in the receptor, rendering it inactive [13], initiate a post–receptor event with a memory [15], or generate an active metabolite with poor permeability [21]. Recently, the first detailed investigations of this issue have revealed evidence for an irreversible mechanism. Specifically, it has been reported that nor-BNI, GNTI and JDTic activate the enzyme c-Jun N-terminal kinase 1 (JNK1, MAPK8), which in turn causes prolonged inhibition of κ signaling [18,22,23]. More recently, it has been reported that κ antagonists with low efficacy towards JNK1 have short durations of action [7].

As a contribution to evaluating these mechanisms, we investigated one of the fundamental questions at issue: are these drugs in fact slowly absorbed and eliminated? We administered nor-BNI, GNTI and JDTic to mice at 10 mg kg^-1^ i.p., as used in a previous study of their timecourses [18]. At various timepoints, the mice were sacrificed, and drug levels in brain and plasma were determined. We also measured physicochemical properties relevant to disposition and depot formation. Specifically, we measured drug binding to plasma proteins and brain homogenate. We also measured octanol/water drug distribution, a standard measure of lipophilicity, at physiologic pH (7.4). As a further test of lipophilicity or hydrophobicity, we measured the water solubility of each compound. Lastly, we performed permeation experiments in cells expressing human permeability glycoprotein (P-gp) to evaluate the rates of active transport and passive diffusion for these compounds across cell membranes.

## Results

### Plasma and brain timecourses

**Figure 2:**
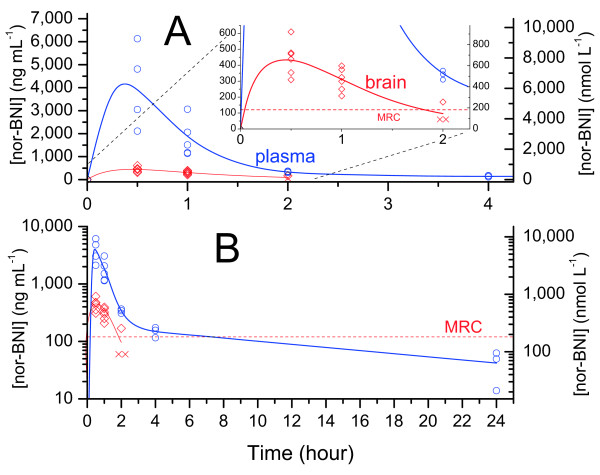
**Nor-BNI levels in plasma and brain in mice over 24 hours (10 mg kg**^**-1**^ **i.p.).** A, linear scale with inset of brain levels; B, log scale [× = MRC÷2 (estimate for points < MRC)].

Plasma and brain levels of nor-BNI are shown in Figure 2; raw data for figures and tables can be found in Additional file 1. Mean plasma nor-BNI was maximal at 30 minutes, then declined significantly within 1 hour (56%, *p* = 0.04). The decline was almost complete within 2 hours (92%). After this, there was a reduction in the elimination rate (see Figure 2B), with an eventual decline of 99% by 24 hours.

Brain uptake of nor-BNI was very low. Standard LC-MS-MS conditions gave lower sensitivity in brain than for a wide range of drugs tested previously [24]. As a result, we obtained accurate brain levels for nor-BNI only up to 1 hour. Nonetheless, no lag was apparent, indicating rapid equilibration between blood and brain. Mean brain nor-BNI was maximal at 30 minutes, and declined significantly within 1 hour (31%, *p* = 0.03). By 2 hours, 2 of 3 samples were below the Minimum Reportable Concentration (MRC) for our assay (120 ng mL^-1^). This permits only an estimate of the initial elimination rate, which is often much higher than the terminal elimination rate. Thus, our data do not establish the terminal elimination rate in brain.

**Figure 3:**
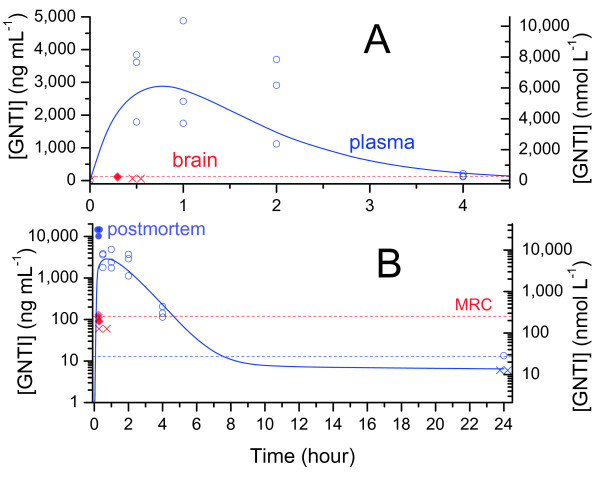
**GNTI plasma and brain levels in mice over 24 hours (10 mg kg**^**-1**^ **i.p.).** Shaded points represent postmortem samples after 39 mg kg^-1^. A, linear scale; B, log scale [× = MRC÷2 (estimate for points < MRC)].

Absorption and elimination of GNTI were somewhat slower (Figure 3). Again however, mean plasma levels were maximal at 30 minutes, and declined by 95% within 4 hours (*p* = 0.01). Viewed on a log scale there was again a marked decline in the elimination rate after 4 hours (Figure 3B). Brain uptake was extremely low. Levels were below MRC (120 ng mL^-1^) at all timepoints, and thus could not be accurately quantified, so the timecourse in brain could not be determined.

**Figure 4:**
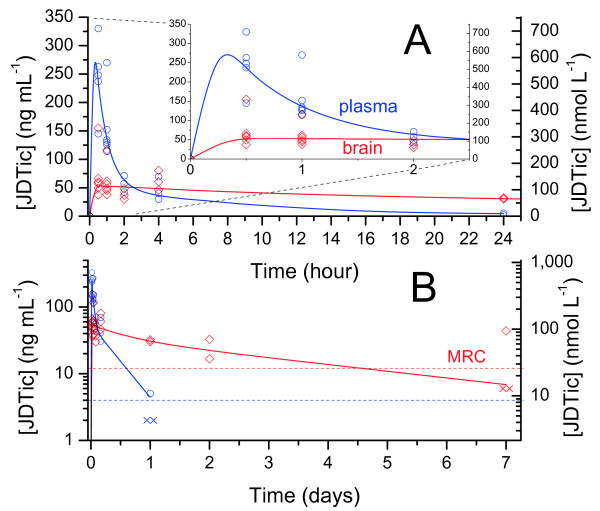
**JDTic plasma and brain levels in mice (10 mg kg**^**-1**^ **i.p.).** A, linear scale over 24 hours with inset of early timepoints; B, log scale over 7 days × = MRC÷2 (estimate for points < MRC).

As with nor-BNI and GNTI, JDTic was rapidly absorbed, reaching peak plasma and brain levels within approximately 30 minutes (Figure 4). Mean plasma concentration declined by 37% within 1 hour (*p* = 0.04), and 77% within 2 hours (*p* = 0.003). Brain levels were markedly lower than plasma, but the drug was very persistent: mean brain JDTic declined by only 56% over 24 hours, and the drug was still detectable at 1 week (Figure 4B). The terminal half-life of approximately 9 days was comparable to the previously reported rate of decline in antagonism [18].

The possibility remains that nor-BNI also persists at lower levels, below our MRC. There have been preliminary reports of the detection of nor-BNI in mouse brain up to 21 days after administration, but details have not yet been reported [25,26]. Nonetheless, our results indicate that the initial elimination rate from brain for nor-BNI is much higher than for JDTic, with brain levels declining by at least 75% within 4 hours. Our results do not permit conclusions about the brain timecourse of GNTI.

### Physicochemical properties

We found that nor-BNI, as the standard salt (dihydrochloride hydrate), showed poor wettability and dissolved slowly. This property may have contributed to the perception that the drug is hydrophobic. In fact, however, solubility was very high: greater than 45 mM for nor-BNI and JDTic, and at least 100 mM for GNTI (Table 1). These values are comparable to the short-acting antagonist naltrexone. By contrast, some lipophilic drugs such as steroids have solubilities below 1 μM, at least 10^5^-fold lower than GNTI [27]. Notably, all of the κ antagonists crystallize as hydrates, whereas most drug salts crystallize as anhydrates [28]. Thus, as measured by both solubility and degree of hydration, the κ antagonists are unusually hydrophilic.

**Table 1:** Physicochemical properties and plasma and brain homogenate binding of nor-BNI, GNTI, JDTic, and naltrexone

	**Nor-BNI**	**GNTI**	**JDTic**	**Naltrexone**
Salt tested	2HCl·H_2_O	2HCl·1.5H_2_O	2HCl·H_2_O	HCl·2H_2_O
Water solubility (mmol L^-1^, 25 °C)	>45	>100^a^	>45	>100^a^
		*mean*		
		*(95% CI)*		
Log_10_D_7.4_	1.5	-0.1	1.8	0.86
octanol:buffer (pH 7.4)	(1.3; 1.8)	(-0.2; 0)	(1.7; 2.0)	(0.82; 0.90)
Fraction unbound (%)				
Plasma	51	28	19	83
	(44; 59)	(24; 31)	(17; 21)	(72; 94)
Brain homogenate	12	*7*	7	47
	(10; 17)	(6; 9)	(6; 8)	(34; 65)

Experiments performed in triplicate (*n* = 3).

^a)^Tocris product information sheet.

The octanol/water distribution coefficient at physiological pH (log D_7.4_), a standard measure of lipophilicity for ionizable compounds, provides further evidence that these drugs are not unusually hydrophobic (Table 1). While more hydrophobic than naltrexone, nor-BNI and JDTic nonetheless fell within the moderate log D_7.4_ range of 1–3, which is optimal for a wide range of pharmacological properties [29]. To put this in context, the short-acting cannabinoid THC has a reported log D_7.4_ value of 7 (10^5^–fold more lipophilic than JDTic) [30]. Interestingly, GNTI proved to be very hydrophilic, with a log D_7.4_ approximately equal to morphine [31]. Thus, none of the κ antagonists possessed exceptionally high or low lipophilicity, and they differ more from each other by these measures than from naltrexone.

### Brain homogenate and plasma binding

We measured drug binding to plasma proteins and brain homogenate, since it is the concentration of unbound drug in solution, rather than total concentration, which governs pharmacological response [32]; also, affinity for brain homogenate provides an experimental test of the membrane depot hypothesis. Each κ antagonist showed greater binding to brain homogenate than did naltrexone, with unbound fractions below 12% (Table 1). However, these values are not exceptional: some CNS-active drugs exhibit unbound fractions below 0.1% in brain homogenate [33].

Recent work indicates that homogenate binding systematically underestimates binding to intact tissue by approximately 3-fold for basic drugs [34]. For theoretical reasons discussed below, the underestimate is expected to be substantially greater for dibasic compounds like the long-acting κ antagonists (Figure 1) [34]. However, since validated results for a set of dibasic drugs are not available, in the calculations that follow we have conservatively assumed in vivo brain binding at least 3-fold greater than the homogenate value.

### Brain uptake

A common measure of brain uptake is *K*_*p,brain*_, or drug exposure in brain relative to plasma. However, *K*_*p,brain*_ values do not accurately predict drug concentrations at the effect site, since they are confounded by tissue and plasma binding [34]. After correction for binding, the relative unbound brain/plasma exposure (*K*_*p,uu,brain*_) gives a much more accurate guide to drug levels in interstitial fluid, as validated against microdialysis [34].

Nor-BNI’s unbound brain/plasma exposure (*K*_*p,uu,brain*_) was extraordinarily low, less than 0.007 (see Table 2). This is comparable to the peripherally-restricted opioid loperamide [35]. While surprising for a centrally-active drug, this value is consistent with the discrepancy between the extremely high potency nor-BNI exhibits in vitro [2] and its low potency in vivo.

**Table 2:** **Pharmacokinetic parameters of nor-BNI, GNTI and JDTic (·2HCl salts, 10 mg kg**^**-1**^ **i.p.), estimated using non-compartmental analysis**

			**Nor-BNI**	**GNTI**	**JDTic**
	*t*_*max*_	h	0.5	0.5	0.5
	*C*_*max,p*_	ng mL^-1^	4,025	3,080	245
	*C*_*max,brain*_	ng mL^-1^	443	< 120	71
	*C*_*max,brain,u*_	nmol L^-1^	< 26	< 6	< 4
	*AUC*_*0–∞,p*_	ng h mL^-1^	6,422	9,486	888
	*AUC*_*0–∞,brain*_	ng h mL^-1^	593	-	10,304
	*V*_*z*_*/F*	L kg^-1^	19	4.4	83
	*CL/F*	L kg^-1^ h^-1^	1.7	1.0	1.1
*AUC0*_*–∞,brain*_/*AUC0*_*–∞,p*_	*K*_*p,brain*_		0.09	–	11.6
*AUC*_*0–∞,brain,u*_/*AUC*_*0–∞,p,u*_	*K*_*p,uu,brain*_		< 0.007	–	< 1.4
*C*_*max,brain*_/*C*_*max,p*_			0.11	0.0085^a^	0.29
*C*_*max,brain,u*_/*C*_*max,p,u*_			< 0.009	< 0.0007^a^	< 0.04

a) From postmortem samples, 13–18 minutes after 39 mg kg^-1^ GNTI·2CF_3CO2H_.

Abbrevations: AUC, area under curve; CL, clearance; C_max_, maximal mean concentration; F, bioavailability; p, plasma; t_max_, time at C_max_; u, unbound (free); Vz, terminal-phase apparent volume of distribution.

Unbound brain exposure for JDTic was much greater than for nor-BNI, up to a possible upper bound of 1.4 (Table 2). This was puzzling given the low peak brain level of JDTic. As an alternate measure, we calculated unbound brain uptake using peak brain and plasma levels. By this measure, unbound uptake of JDTic was very low, < 0.04, in marked contrast to the exposure measure. For nor-BNI, the two methods gave concordant results. Calculations based on exposure may be misleading for JDTic due to the extraordinarily large difference in elimination rates between brain and plasma. Comparison of drug concentrations in brain and plasma clearly establishes low unbound uptake for this drug. Further evidence on this question can be found in the results of our active transport experiments below.

Brain exposure could not be quantified for GNTI at 10 mg kg^-1^. In an attempt to obtain quantifiable brain levels we administered higher doses, which unexpectedly proved lethal, as described in the next section. At 10–18 minutes after GNTI bis-trifluoroacetate (39 mg kg^-1^), postmortem brain and plasma samples revealed a very high mean plasma concentration of 13,200 ng mL^-1^, but a mean brain level (112 ng mL^-1^) still just below the MRC (120 ng mL^-1^). It should be noted that the low brain levels at this early timepoint may reflect incomplete equilibration. Nonetheless, GNTI clearly exhibited extremely low brain uptake in all of these experiments.

**Figure 5:**
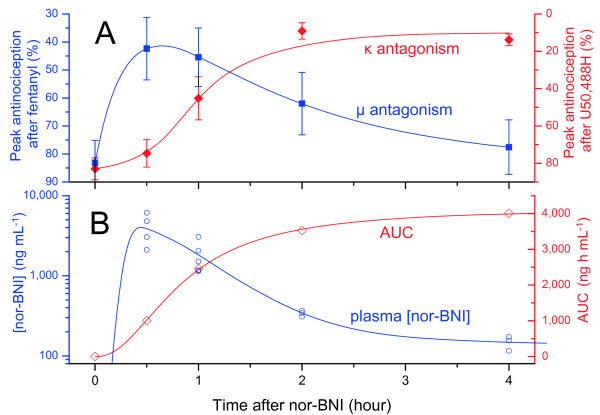
**A, Reported timecourses of μ and κ antagonism over 4 hours after nor-BNI (mean ± SEM, 20 mg kg**^**-1**^ **s.c., mice**[10]); B, nor-BNI plasma levels and mean area under curve (AUC) over the same period.

Even these very low uptake values represent conservative upper bounds, and the true values are likely to be lower still. We have chosen a minimal value for the underestimate of tissue binding using homogenate, as noted above, and the true binding is likely to be greater. Also, we have not corrected for residual plasma in brain. The brain is estimated to contain approximately 1–3% blood by volume, which can make a substantial contribution to total brain drug content for drugs with low uptake [32]. However, correction for this is complex, and to our knowledge the required parameters have not yet been established in mice. This correction would further reduce the estimated brain uptake. Consistent with this, note that we estimate peak unbound brain levels at 30 minutes of up to 4 nM for JDTic and 26 nM for nor-BNI (Table 2). Given that the binding affinities and potencies of these drugs in vitro are consistently sub-nanomolar [2], these concentrations would be expected to produce near-maximal receptor occupancy, and marked increases in the ED_50_ of κ opioids. As noted above, however, negligible κ antagonism is observed at 30 minutes (Figure 5). This suggests that the true unbound brain concentrations are considerably lower.

In summary, our evidence suggests that all three long-acting κ antagonists exhibit extremely low brain uptake relative to other centrally-active drugs, but accurate quantification will require further research.

### Fatalities after GNTI

After 30 mg kg^-1^ GNTI dihydrochloride, all three mice unexpectedly became ataxic, followed by convulsions and death within 11 minutes of injection. To confirm that this was not due to an impurity, the experiment was repeated with an equimolar dose of a different salt from a different supplier (bis-trifluoroacetate, 39 mg kg^-1^). Again, all three mice died within 18 minutes. At 10 mg kg^-1^, GNTI caused no fatalities in 15 mice monitored for at least 30 minutes. This difference was of extremely high statistical significance (*p* < 0.0001 by Fisher’s Exact test, two tailed). Even at 100 mg kg^-1^, no fatalities occurred after nor-BNI (3 mice, *p* = 0.01 vs. GNTI) or JDTic (6 mice, *p* = 0.002). Only mild behavioral effects, such as hiccup-like spasms and shivering, were observed. The lower toxicity of these two drugs, despite their comparable potency as κ antagonists, suggests that GNTI's toxicity may involve some form of efficacy or a different target.

### Apparent volume of distribution

In addition to the direct tests of binding to plasma and brain homogenate, our results yield an indirect measure of tissue affinity. It is notable that at the same dose, JDTic gave maximal plasma concentrations less than 10% of those seen after nor-BNI and GNTI (Table 2). This might reflect lower absorption into plasma, higher affinity for tissue relative to plasma, or both. This property can be quantified using the volume of distribution *(V)*.

Based on the above timecourse and binding data, pharmacokinetic parameters were calculated using non-compartmental analysis (Table 2). Apparent terminal-phase volumes of distribution *(V*_*z*_/*F)* for GNTI (4.4 L kg^-1^) and nor-BNI (19 L kg^-1^) were unremarkable in comparison to the values of *V* for a wide range of other drugs determined previously in rats (0.1 – 73 L kg^-1^) [24]. The volumes of distribution for GNTI and nor-BNI therefore appear to be low or moderate relative to other CNS-targeted drugs, suggesting moderate tissue affinity relative to plasma. This is consistent with the brain homogenate binding we observed.

However, the apparent volume of distribution for JDTic was extremely high (83 L kg^-1^). While this might be due to low bioavailability *(F)*, it is also consistent with a very high value for *V* – that is, very high affinity for tissue relative to plasma. JDTic’s negligible rate of elimination from brain, but not plasma, provides strong confirmation of high tissue affinity. This is in marked contrast to the compound's moderate affinity for brain homogenate. Given that membranes do not represent a plausible basis for this affinity, we propose an alternative in the discussion section.

### Membrane permeation and efflux

The membrane permeabilities of these drugs were evaluated using monolayers of cells expressing human P-gp. All three of the long-acting antagonists showed extremely low passive permeability, up to 150-fold lower than naltrexone, as measured by apical to basolateral flow rates (Table 3). No active transport of nor-BNI or GNTI was detectable, but JDTic showed a very high efflux ratio. Consistent with previous reports [36], the known P-gp substrate loperamide also showed high efflux, but naltrexone did not. These results suggest that JDTic is a P-gp substrate, but that nor-BNI and GNTI are not.

**Table 3:** Mean permeation rates and efflux ratios in LLC-PK1-MDR1 cell monolayers

			**Nor-BNI**	**GNTI**	**JDTic**	**Naltrexone**	**Loperamide**
P_app_	(A → B)	nm s^-1^	2	4	5	310	12
P_app_	(B → A)	nm s^-1^	3	6	240	410	470
Efflux ratio (B → A/A → B)			1.5	1.5	48	1.3	38

A = apical, B = basolateral. Experiments performed in duplicate (*n* = 2).

## Discussion

### Absorption rate and transient side effects

As noted above, nor-BNI plasma levels peaked early and declined rapidly. Interestingly, as seen in Figure 5, the reported timecourse of μ antagonism fits this plasma timecourse closely. The rise in κ antagonism is diametrically opposed to the decline of plasma levels between 30 minutes and 4 hours. Interestingly, however, a close correspondence is apparent to cumulative exposure (area under curve, AUC). Both κ antagonism and AUC exhibit a low initial rate of increase when drug level is low, a maximal rate of increase at ~30 minutes when drug level peaks, then a gradual taper to a plateau as the drug is eliminated. It should be noted that these antagonism data were obtained after subcutaneous rather than intraperitoneal administration. Nonetheless, these routes give very similar absorption rates: analgesia peaks 15–30 minutes after administration of morphine to mice by either i.p. [37] or s.c. routes [38]. The striking correspondence between plasma concentrations and μ antagonism in Figure 5 suggests that the same is true of nor-BNI.

**Figure 6:**
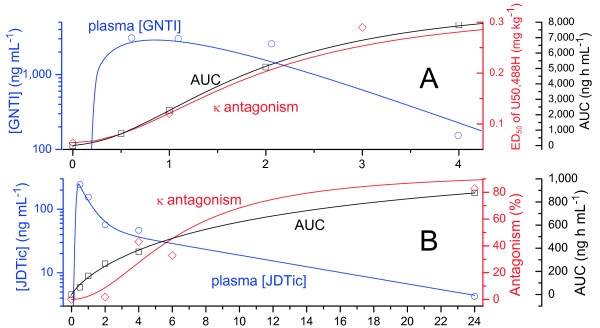
**Early mean plasma levels and AUC compared to the reported timecourses of κ antagonism for GNTI(A: rhesus monkeys, 1 mg kg**^**-1**^** i.m. [**[14]**]) and JDTic (B: mice, i.g. [**[15]**]).**

The same pattern is evident for GNTI in Figure 6A. As noted in the Background section, GNTI induces transient sedation. In mice (i.p.), sedation was maximal within 20 minutes and lasted less than 3 hours [39]; in rhesus monkeys (i.m.), rapid onset and a duration of “several hours” was reported [14]. Thus, despite the different routes of administration, the timecourse of that effect in both species closely approximates the plasma timecourse observed here. A close resemblance is also evident again between the delayed onset of κ antagonism and our cumulative AUC data.

As with GNTI, JDTic reportedly does not cause transient antagonism of μ opioids [19]; nor are we aware of any reports of other transient side-effects. However, a close correspondence between the onset of κ antagonism and AUC is again apparent (Figure 6). Notably, due to the slower elimination of JDTic compared to nor-BNI and GNTI, the rise in AUC was much slower, matching the much slower onset of antagonism.

These antagonism data were obtained after intragastric (i.g.) administration of JDTic, which may delay absorption compared to the intraperitoneal route we employed. However, absorption after i.g. administration in rodents is typically rapid. Drugs with diverse absorption rates in vitro attain peak plasma levels 15–45 minutes after i.g. administration to rats [40]. Similarly, the effects of opioid antagonists including naloxone and naltrexone are maximal, or non-significantly below maximal, within 30 minutes of i.g. administration in mice [41] and rats [42]. Maximal analgesia after i.g. morphine is delayed by 30 minutes or less compared to i.p. administration [37]. Thus, intragastric administration cannot plausibly account for the delay in onset of JDTic, which is substantially greater than 6 hours (Figure 6B). Furthermore, JDTic exhibits equally slow onset after subcutaneous administration in rats [15]. At equal doses, the effects of nor-BNI were equal to, or greater than, those of JDTic over the first five hours. But after a week, JDTic's effects had substantially increased, while those of nor-BNI were unchanged or reduced, leaving JDTic consistently more effective [15]. This confirms that maximal κ antagonism after JDTic is delayed by at least several hours compared to nor- BNI, despite the equally rapid absorption we observed.

### Elimination rate and duration of action

**Figure 7:**
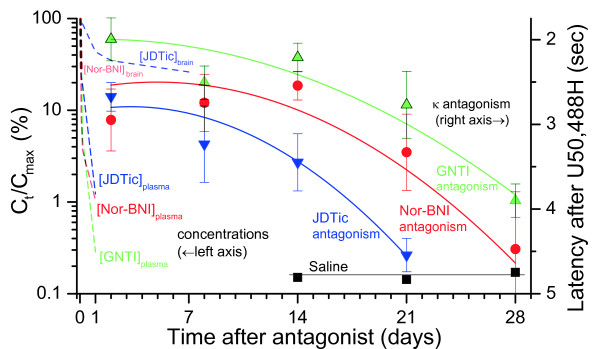
**Elimination of nor-BNI, GNTI and JDTic from brain and plasma compared to the reported durations of κ antagonism at the same dose (mean ± 95% CI, 10 mg kg**^**-1**^**i.p. in mice, tail flick assay**[18]).

Plasma levels of nor-BNI, GNTI, and JDTic declined rapidly, with mean concentration falling at least 80% below peak within 4 hours in all cases, and at least 98% below peak at 24 hours. By contrast, reported κ antagonism is still maximal at 48 hours, and declines gradually over 3–4 weeks at the same dose (Figure 7). However, plasma levels do not always accurately predict concentrations in the brain. For instance, plasma levels of haloperidol in rats decline rapidly (undetectable within 2 days), while brain concentration takes 3 weeks to reach the same level, corresponding closely to the decline in antagonism [43]. Our data do not establish the rate of elimination from brain for GNTI, but indicate rapid initial elimination for nor-BNI (in contrast to the long duration of antagonism).

**Figure 8:**
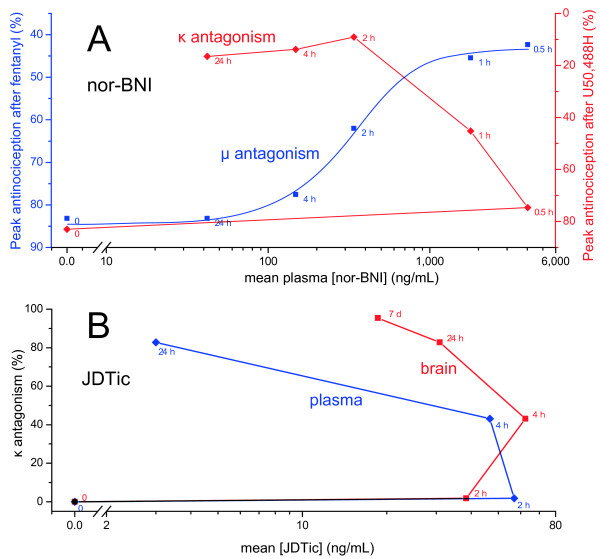
**Concentration-effect relationships derived from Figures 5 and 6.** A: Plasma nor-BNI concentrations against reported μ and κ antagonism at the indicated times (mice, 20 mg kg^−1^ s.c. [10]). B: Plasma and brain JDTic concentrations against reported κ antagonism (mice, i.g. [15]).

For JDTic, however, the rates of decline in brain concentration and antagonism were comparable. Thus, it would seem more plausible to attribute JDTic’s long duration of action to persistence of the drug in brain than for nor-BNI and GNTI. This will be discussed further below. It is important to note, however, that this proposal leaves delayed onset unexplained. Indeed, the lag (or hysteresis) between peak drug level and peak antagonism is many hours longer for JDTic than nor-BNI and GNTI (compare Figures 5 and 6). A common and intuitive way to analyze hysteresis is to plot drug concentration versus effect over time. In Figure 8, our concentration data for nor-BNI and JDTic are plotted against the previously reported antagonism data from Figures 5 and 6.

Competitive, reversible drug effects show a sigmoidal relationship with drug concentration in vitro. In vivo, opioids with diverse physicochemical properties and pharmacokinetic parameters show sigmoidal brain concentration-effect relationships [37,38]. As seen in Figure 8A, plasma levels of nor-BNI exhibit a strong sigmoidal relationship with μ antagonism, consistent with such a competitive, reversible mechanism of action.

Drugs with slow blood–brain equilibration show a weaker correlation, with the points forming a ‘hysteresis loop’ [37,38], but a strong positive correlation remains between concentration and effect. By contrast, the correlation between plasma [nor-BNI] and κ antagonism is strongly negative; that is, maximal plasma concentrations coincide with minimal antagonism and vice versa. Brain concentrations have not been plotted due to the small number of points available, but the same discrepancy is apparent in Figure 2. For JDTic, plasma concentration and κ antagonism were also negatively correlated (Figure 8B). Despite slower elimination from brain, the correlation with brain concentration was also negative. There were insufficient common timepoints available to plot a similar curve for GNTI, but it is evident from Figures 6 and 7 that the same negative correlation holds.

### Resemblance to irreversible antagonists

Thus, for all three of these drugs there is a strongly negative correlation between concentration and κ antagonism over most of the timecourse, unlike the positive correlation characteristic of reversible, competitive drug action. Similar discrepancies are seen with irreversible antagonists. For example, β-funaltrexamine (β-FNA) binds covalently to the μ opioid receptor, as recently confirmed by crystal structure [45]. This results in prolonged μ antagonism in vivo [45], which can exhibit delayed onset both in vitro [46] and in vivo [47]. However, β-FNA is also a reversible κ agonist*,* and unlike μ antagonism this effect is of rapid onset and brief duration in vivo [45].

While reversible effects are a function of drug concentration, irreversible effects persist after the drug is washed out [48]: they are thus a function of prior exposure rather than concentration. As a corollary to this, irreversible effects continue to rise as long as drug and substrate are present, resulting in delayed onset [48]. This severe hysteresis in the concentration-effect relationship, where maximal effect can coincide with minimal concentration and vice versa, is a noteworthy similarity between the long-acting κ antagonists and irreversible antagonists. The occurrence of transient side-effects is another striking similarity between β-FNA, nor-BNI and GNTI.

There are also important differences, however. Nor-BNI, GNTI, and JDTic all bind reversibly in vitro, and lack reactive functionalities capable of forming a covalent bond to the receptor [2]. For JDTic, this has recently been definitively confirmed by the crystal structure in complex with the κ opioid receptor [49]. Consistent with this, all three drugs produce surmountable antagonism [2,3]. Insurmountable antagonism (a reduction in maximal response to agonists) is a defining characteristic of irreversible antagonists. It is important to note, however, that insurmountable antagonism is not always observed – in systems with high receptor reserve, irreversible antagonists can produce surmountable antagonism [50].

### Resemblance to agonist-induced desensitization

The hysteresis typical of irreversible drugs is also seen when drugs bind reversibly, but induce irreversible effects. Daily doses of the κ opioid U50,488H induce tolerance, such that doses which initially produced near-maximal analgesia are without effect by the fifth day [51]. This desensitization is believed to be mediated by phosphorylation of the receptor: while the drug itself binds reversibly, it promotes a covalent modification which inhibits signaling. This process shares some of the characteristics of an irreversible antagonist, notably a very long duration (two weeks after moderate doses of U50,488H) [51], and slow onset. Due to the very slow rate of resensitization, the process is cumulative, and rises with each dose rather than falling with plasma drug levels, resulting in gradual onset over several days. By contrast, the reversible effects of the drug (such as analgesia and sedation) are of rapid onset and brief duration.

As with irreversible antagonists, desensitization may cause insurmountable antagonism, but this is not always observed [50]. Repeated administration of κ opioids can produce either surmountable or insurmountable desensitization [52]. Similarly, desensitization may reduce maximal binding (B_max_) of radioligands, rather than affinity (*K*_*d*_*),* but the opposite effect has also been reported, depending on experimental conditions [52].

### Explanation of hysteresis by JNK-mediated desensitization

Our results are consistent with recent reports that long-acting κ antagonists activate c-Jun N-terminal kinase 1 (JNK1), which in turn inhibits signaling [18,22]. As noted above, resensitization at κ is very slow, and JNK1-mediated desensitization might therefore be expected to be long-lasting. Since persistent desensitization will be cumulative, we propose that it may display delayed onset, as with agonist-induced desensitization and irreversible antagonism.

Specific mechanisms for the observed transient side effects have not yet been proposed. However, any reversible effects of the drugs would be expected to be a function of drug levels, as seen with β-FNA. The effects might result from reversible interactions with other targets, or from reversible effects upon specific κ signaling pathways. The JNK1 hypothesis thus provides a parsimonious explanation for the occurrence of both delayed, prolonged antagonism and rapid, transient side-effects.

However, our finding that JDTic is much more slowly eliminated than nor-BNI from brain is more difficult to explain by this hypothesis, given their similar durations of action. If both compounds induce prolonged desensitization, the continued presence of JDTic in brain would be expected to continue activating JNK1, resulting in a longer duration of action. One explanation could be desensitization of the JNK1 pathway itself.

It was recently found that when nor-BNI was administered twice over 72 hours, only the first dose activated JNK1 [7]. Alternatively, total brain content of JDTic may not accurately reflect unbound drug levels at the receptor. This would be the case, for instance, if the drug is sequestered within cells, a possibility discussed below.

### Explanation by formation of active metabolites

Another proposed mechanism for nor-BNI‘s duration of action was the generation of active metabolites with poor permeability, which would therefore persist in the brain [21]. While this hypothesis cannot be excluded using our results, it would require potent metabolites with much slower elimination than the parent drug in each case. However, opioid metabolites are overwhelmingly inactive or less potent than the parent compound, and rapidly eliminated [53]. Moreover, this hypothesis does not account for the delayed onset of κ antagonism, and thus leaves a key aspect of the overall hysteresis unaccounted for.

### Explanation by slow absorption and distribution

Contrary to early proposals that nor-BNI might enter the brain slowly due to poor membrane permeability [20], plasma and brain levels of all three drugs were maximal at the first timepoint tested (30 minutes). This is typical: drugs with a wide range of physicochemical properties, including those with poor permeability or low uptake, typically show very little lag between peak blood and brain levels [38].

**Figure 9:**
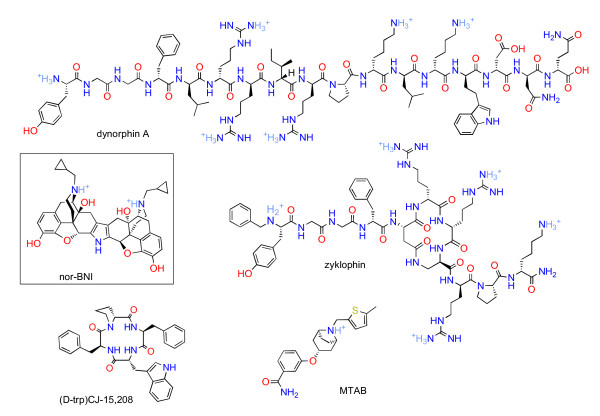
Structures of nor-BNI, short-acting κ antagonists and dynorphin A, to the same scale.

Moreover, nor-BNI exhibits delayed onset even after i.c.v. administration, when permeation of the blood–brain barrier is not required. To account for this, it was speculated that the bulkiness of the dimeric drug (relative to the monomer naltrexone) might result in slow diffusion to the site of action [20]. However, the endogenous κ opioid dynorphin A is much larger than nor-BNI (Figure 9), yet exhibits rapid onset after i.c.v. administration, with peak effects in under 15 minutes [54]. Also shown in Figure 9 is zyklophin, recently reported to cause κ antagonism of rapid onset and short duration [5]. Zyklophin is also much larger than nor-BNI. Other new short-acting κ antagonists are shown in Figure 9: CJ-15,208 is of comparable size to nor-BNI [55], while MTAB is smaller [4]. Since both larger and smaller κ ligands exhibit rapid onset, nor-BNI’s delayed onset cannot be convincingly explained by its size.

Proposals based upon slow absorption and distribution also fail to account for the conflicting timecourses of the transient effects and κ antagonism noted above. If the delayed onset and long duration of κ antagonism were due to slow uptake and elimination, transient effects of rapid onset and brief duration would not be expected.

### Explanation by membrane depot formation

It has been proposed that nor-BNI’s long duration of action might result from dissolution in cell membranes, creating a depot from which the drug would slowly diffuse, maintaining drug levels at the receptor [13]. However, the assumption that any of these drugs is highly lipophilic is unsupported by theory or evidence. For each of these drugs, the predominant species at physiologic pH is doubly charged (Figure 1 and Table 1), with numerous hydrogen bond donors and acceptors, features which increase hydrophilicity. Consistent with this, all three drugs exhibit high water solubility, and an unusually strong tendency to crystallize as hydrates. Also consistent with this, JDTic binds to the κ opioid receptor via a network of eight hydrogen bonds, involving five water molecules [49].

The octanol/water distribution values of nor-BNI and JDTic are only moderately lipophilic, while GNTI is very hydrophilic. Thus, the κ antagonists are not highly lipophilic by any of the measures we examined, and in fact are unusually hydrophilic by several measures. These data do not support the hypothesis that these drugs would show a stronger tendency than other opioids to accumulate in cell membranes or other lipids. The moderate brain homogenate binding we observed provides further evidence that these drugs do not bind strongly to cell membranes, or indeed to any other cellular component.

A second assumption of this hypothesis, that a membrane depot would replenish effective drug levels at the receptor for weeks or months, is also questionable. (D-trp)CJ-15,208 is neutral (Figure 9), and so hydrophobic that a 50% DMSO vehicle was required for injection, but nonetheless has a duration of action under 24 hours [55]. THC is extremely lipophilic, with correspondingly high affinity for membranes [30], but has an even shorter duration of action.

The depot hypothesis has been tested experimentally: Bruchas et al. reported that pre-administration of the rapidly-eliminated antagonist naloxone (30 mg kg^-1^ i.p.) blocked the effects of nor-BNI on subsequent days, suggesting that nor-BNI does not persist at effective levels longer than naloxone [18]. Paronis et al. did not observe such protection by naltrexone, but the drugs were given by different routes of administration [21].

Subcutaneous naltrexone may not have produced sufficient central levels to counteract intra-cisternal nor-BNI. Also, the dose of naltrexone used (10 mg/kg) may have been insufficient. As noted there, even very high doses of competitive antagonists (100 mg kg^-1^ naloxone) do not achieve full protection against irreversible antagonists [21], and the same may conceivably be true for irreversible effects of competitive drugs.

### JDTic may accumulate in lysosomes

JDTic’s high apparent volume of distribution, and slow elimination from brain, suggest very high tissue affinity. The decline in the plasma elimination rate after 4 hours also suggests a non-plasma compartment with slow elimination. However, the above evidence indicates moderate affinity not only for membranes, but for all components of brain homogenate. Thus, JDTic's tissue affinity appears to depend upon membrane integrity. Also, given its extraordinary persistence in brain despite P-gp-mediated efflux, the drug appears to be isolated from the extracellular fluid and blood–brain barrier. Taken together, this evidence suggests sequestration of JDTic within cells.

One common mechanism for sequestration of basic compounds is trapping within acidic compartments of the cell (lysosomes). Acidic conditions protonate basic compounds, and the resulting charged species is less membrane-permeable. Membrane-permeable basic drugs therefore enter lysosomes more readily than they leave, and become trapped [56]. On average, basic drugs show approximately 3-fold higher affinity for intact cells than for homogenate [34]. Acidic drugs show a slight bias in the opposite direction. The discrepancy is not seen for neutral drugs, and can be abolished by inhibiting lysosomal uptake [34]. The compounds which accumulate to the greatest degree are dibasic, especially when both bases are weak (pKa ~8): the double protonation results in a much greater reduction in permeability [56].

JDTic features two weak bases and optimal lipophilicity for membrane permeation (Figure 1 and Table 1). JDTic might therefore be expected to accumulate in lysosomes on theoretical grounds. This would reconcile JDTic’s apparently high affinity for intact tissue with its low affinity for homogenate, since lysosomal trapping requires membrane integrity. While we have no direct evidence for this, an analogy can be made with the opioid desmethyl-loperamide, whose pharmacokinetic profile is strikingly similar to that of JDTic [57]. Despite very low brain uptake and rapid clearance from plasma, the drug is very persistent in brain, from which it cannot be displaced by opioid antagonists. Desmethyl-loperamide’s low brain uptake is due to P-gp efflux, while its ultra-low brain elimination rate and high tissue affinity are due to lysosomal trapping [57]. This drug thus shows many similarities to JDTic, supporting the conclusions that JDTic is also a P-gp substrate which is subject to lysosomal trapping.

Like JDTic, nor-BNI features two weak bases and optimal lipophilicity, and would therefore also be expected to accumulate in lysosomes (Figure 1 and Table 1). Surprisingly, however, we found no evidence for this: nor-BNI showed a lower apparent volume of distribution, and a much higher initial elimination rate from brain. However, the drug may persist below our minimum reportable concentration, and the decline in elimination rate after 2 hours is again consistent with slow elimination from a non-plasma compartment. As noted above, there have been preliminary reports of the detection of nor-BNI in mouse brain up to 21 days after administration [25,26]. Given the moderate affinity we observed for brain homogenate, this would again be consistent with lysosomal trapping, but not accumulation in cell membranes. GNTI exhibits much lower lipophilicity, and features one weak base along with a strongly basic guanidino group, whose protonation state does not vary appreciably within the physiological pH range (Figure 1). GNTI would therefore be expected to show a smaller decrease in permeability within lysosomes; and indeed, GNTI’s low apparent volume of distribution is consistent with lower tissue affinity than JDTic.

Not all of our evidence supports this hypothesis unambiguously. The very low passive permeability each of these drugs exhibits in vitro (Table 3) suggests poor membrane permeability, which would reduce lysosomal accumulation. However, lyososomal trapping can reduce apparent permeability in these experiments [58], so additional sources of evidence would be needed to resolve this question.

Interestingly, a number of short-acting κ antagonists have been reported recently, none of which are dibasic (Figure 9). CJ-15,208 is neutral [55], while MTAB and others are monobasic [4]. The peptide zyklophin [5] features five strongly basic residues. Thus, these compounds would be expected to accumulate to a much lower degree in lysosomes, or not at all. The correlation is not perfect: recent work has revealed three dibasic JDTic analogues with short durations of action [7]. Nonetheless, given that all firmly established long-acting κ antagonists to date are dibasic, this remains an intriguing topic for further research.

### Active transport and brain uptake

Our in vitro permeation results, discussed above, indicate that JDTic is a P-gp substrate, with active efflux comparable to peripherally-restricted opioids. This is consistent with the low brain uptake we determined. Our results are also consistent with recent preliminary results indicating extremely low unbound brain uptake of nor-BNI and JDTic in rats [4]. In that study, low uptake of nor-BNI was confirmed by brain microdialysis, which is not confounded by plasma drug content.

Nor-BNI and GNTI did not act as substrates of human P-gp in our in vitro assays. Nonetheless, both of these drugs exhibited even lower unbound brain uptake than JDTic, which suggests that they are also subject to active efflux in mouse brain. This discrepancy might reflect differences between human and mouse P-gp (MDR-1a). Alternatively, these drugs may be substrates for another of the many transporters expressed at the blood–brain barrier [59].

### Questions for future research

For future work, a number of interesting questions remain unresolved, and other new questions are raised by these results. A more sensitive analytical method could establish whether nor-BNI and GNTI persist in brain at low concentrations, but would not distinguish bound from unbound drug. This problem could be overcome by the use of in vivo brain microdialysis, which would give the timecourses in extracellular fluid, unconfounded by plasma levels or tissue binding. Intracellular trapping could be evaluated by comparing drug binding in brain slices versus homogenate, which would also allow evaluation of lysosomal uptake and other mechanisms [34]. If tissue uptake could be reduced, for instance by inhibiting lysosomal uptake, the effect on the drugs’ timecourse in vivo would be informative. A clear understanding of the unusual properties of these drugs will be important as members of this class advance toward the clinic.

## Conclusions

Contrary to previous speculations, nor-BNI, GNTI and JDTic were rapidly absorbed and eliminated from plasma after intraperitoneal administration. This timecourse is in striking contrast to the slow onset and ultra-long duration of the κ antagonism they produce, but coincides well with transient side-effects. Thus, the transient effects are positively correlated with plasma concentration, consistent with a competitive mechanism of action. By contrast, κ antagonism is negatively correlated with plasma concentrations, but positively correlated with exposure. Both of these correlations are more consistent with an irreversible mechanism of action such as JNK1-mediated desensitization.

Our results indicate that JDTic is a P-gp substrate, but that nor-BNI and GNTI are not. However, the extremely low brain uptake of these drugs suggests that they may be substrates of another transporter. Our findings that these drugs are relatively hydrophilic by several measures suggest that they will not form a membrane depot. Their modest affinities for brain homogenate also argue against other forms of nonspecific tissue binding. JDTic’s extremely slow elimination from brain, despite low affinity for homogenate and active efflux, suggests intracellular trapping. We propose lysosomes as a plausible site for this entrapment, but no direct evidence is yet available. JDTic’s persistence in brain may contribute to its long duration of action, but this cannot account for its ultra-slow onset, nor for the even longer duration of action exhibited by nor-BNI.

## Materials and methods

### Animals

Male Swiss-Webster mice (22–24 g, Crl:CFW(SW), Charles River. Laboratories, Wilmington MA, USA) were housed under a 12 h light/dark cycle in clear polycarbonate boxes (three per box) with pine chip bedding, nesting pads, and unrestricted food and water. Experiments were performed between 10 am and 6 pm. Mice were not used in other experiments, or exposed to other drugs, before testing began. This study followed the recommendations of the Institute for Laboratory Animal Research [60], and has been reported according to the ARRIVE guidelines [61]. The protocol was approved by McLean Hospital Institutional Animal Care and Use Committee (protocol 09-5/2-16)

### Drugs

GNTI∙2HCl∙1.5H_2_O: Tocris Bioscience, Ellisville MI (batches 4B/91591 and 4B/94133, >99.4% purity). GNTI∙2CF_3_CO_2_H∙2.6H_2_O: Sigma-Aldrich, St Louis MI (lot 096 K4605, 97.6% purity). JDTic∙2HCl∙H_2_O: F. Ivy Carroll, Research Triangle Institute, NC. Naltrexone∙HCl∙2H_2_O: Tocris Bioscience (batch 5B/93329). Nor-BNI∙2HCl∙H_2_O: Tocris Bioscience (batches 8A/90732 and 9A/93084). Vincristine: Sigma-Aldrich, St Louis MI.

### Other materials

Medium 199 and heat-inactivated fetal bovine serum (FBS): Invitrogen (Carlsbad, CA). Non-collagen-coated transwell plates (0.4 μm pore size, 0.7 cm^2^ surface area): Millipore Corporation (Billerica, MA). Bovine serum albumin (BSA), ethylenediaminetetraacetic acid (EDTA), 1-octanol and phosphate-buffered saline (PBS, pH 7.4): Sigma-Aldrich, St Louis MI.

### Drug administration

Doses are reported by weight of the specified salt. Intraperitoneal injections were administered in distilled water vehicle (10 mL kg^-1^). Body mass was measured to ±0.1 g. Some values were inadvertently measured on a low-precision balance (± 2.5 g); these timepoints were replicated at ±0.1 g. Comparison of results from these replicates revealed no statistically significant difference, so all results were pooled for analysis.

### Sample preparation

Mice were sacrificed by cervical dislocation, then decapitated for collection of brain and blood samples. Trunk blood was collected in 1.5 mL polypropylene micro-centrifuge tubes, shaken with a few crystals of EDTA and cooled in ice-water. Samples were centrifuged at 14,000 rpm (revolutions per minute) for 15 minutes at 3 °C, then stored in glass sample vials at −20 °C until analysis. Brains were removed and immersed for 30 seconds in 2-methylbutane over dry ice, then stored in polypropylene tubes at −20 °C until sample preparation. Sample tubes were thawed in ice-water, distilled water was added (2:1 by weight) and the brain homogenized with a probe sonicator for 3 minutes over ice-water (Fisher Sonic Dismembrator 300 at 35% power). The probe was rinsed with ethanol and distilled water between samples. Brain homogenate was stored in glass sample vials at −20 °C until analysis by LC-MS-MS.

### LLC-PK1-MDR1 cell permeation and efflux

Pig kidney epithelial cells (LLC-PK1) were obtained from American Type Culture Collection (Manassas, VA). Transfection with human MDR1 gene was conducted at Amgen (Thousand Oaks, CA). Bi-directional permeation assays were performed as described previously [62]. Briefly, cells were grown in Medium 199 supplemented with 10% FBS in the presence of vincristine (640 nM). Cells were seeded onto Transwell filter membranes (0.4 μm pore size, surface area = 0.7 cm2) at a density of 200,000 cells/well. Compound incubations (in duplicate) were performed 5 days post-seeding. To determine efflux, compounds were tested at 5 μM in the presence of 0.1% BSA. Transport studies were conducted at 37 °C in a humidified incubator with shaking (70 rpm) for 120 min. Samples were analyzed by LC-MS-MS.

### Octanol-buffer distribution

Phosphate-buffered saline (PBS, pH 7.4) and 1-octanol were mutually saturated by vigorous stirring at room temperature for 18 h, and then separated. Drug solutions of 0.1 mg mL^-1^ in PBS were prepared, and 500 μL aliquots were added to 1.5 mL micro-centrifuge tubes with an equal volume of 1-octanol. Three samples were vortex-mixed for 1, 10 and 30 minutes respectively, then centrifuged at room temperature (12,000 rpm for 15 minutes). Aliquots of the upper octanol layer were removed by pipette. A disposable syringe was inserted while bubbling air through the upper layer, an aliquot of the lower PBS layer withdrawn, and the needle removed before transferring. Samples were stored in glass sample vials at −20 °C until analysis by LC-MS-MS. Since no trend was evident with mixing time, results from the different mixing times were pooled.

### Water solubility

Distilled water was added slowly by microsyringe to 10–15 mg of drug in a glass vial, swirling until no solid was visible.

### Determination of drug concentrations

Drug concentrations (ng mL^-1^) refer to the free base, and were measured by liquid chromatography-tandem mass spectrometry (LC-MS-MS) as described previously [24]. Briefly, samples were shaken with 3 volumes of methanol and centrifuged at 3000 g. Aliquots of the supernatants were diluted with 5 volumes of water containing an appropriate internal standard. Extracts from in vitro and in vivo experiments were analyzed by multiple reaction monitoring on an API3000 or API4000 LC-MS-MS system with electrospray ion source, controlled and analyzed using the Analyst software package (Applied Biosystems, Foster City, CA). Chromatography was conducted on a Sprite Armor C18 analytical column (20 × 2.1 mm, 10 μm particle size, Analytical Sales and Products, Pompton Plains, NJ) with a 0.5 μm PEEK guard filter. Compounds were eluted with a gradient from 2% to 95% acetonitrile in water, both containing 0.1% formic acid. Compounds were detected in positive ion mode, tuned to the mass transition with the largest intensity. Analyte concentrations were determined by comparison of analyte:internal standard peak area ratios to those of standards prepared in the appropriate matrices.

### Brain homogenate and plasma binding

Unbound fractions in brain homogenate and plasma were determined by ultracentrifugation as described previously [24]. Briefly, drug-spiked matrices (5 μM drug in plasma or brain homogenate) were centrifuged at 600,000 g for 5 h at 37 °C (plasma) or 5 °C (brain). Aliquots of the middle (water) layer were added to an equal volume of blank matrix and extracted with 5 volumes of acetonitrile containing internal standard. Aliquots of the original spiked matrix were mixed with an equal volume of blank matrix and 2 volumes of plasma ultrafiltrate (plasma) or phosphate-buffered saline (tissue) and extracted with 10 volumes of acetonitrile. Extracts were centrifuged and analyzed by LC-MS-MS. Fraction unbound was calculated from the ratio of concentration in the water layer to that in the original spiked matrix, corrected for dilution as described previously [24]. Based on previous results for basic drugs, in vivo free fraction in brain was assumed to be at least 3-fold lower than the in vitro free fraction in brain homogenate [34].

### Data analysis

For calculation of mean concentrations, the earliest values below the MRC were substituted by MRC ÷ 2, as recommended elswhere [63]. Pharmacokinetic parameters were calculated from mean concentrations using PKSolver [64], employing non-compartmental analysis for extravascular administration. Curves in figures were fitted using Origin 8.5 (OriginLab, Northampton, MA) using 2-compartment models. Confidence intervals and two-tailed *p* values (unpaired *t* test) were calculated using Graphpad Quickcalcs [65]. One outlier was identified using Grubbs' test (*p* < 0.01) and excluded from the analysis; see Additional file 1.

### Extraction of published data

Where data were not given in tables, values were extracted from graphs using Engauge Digitizer [66].

## Competing interests

CB, BMC and WAC hold US patents on the use of κ ligands to treat mood disorders (6,528,518 and 7,629,475). FIC holds a US patent on the preparation and therapeutic use of JDTic (6,974,824).

## Author contributions

TAM processed samples, collected plasma samples, performed distribution assays, proposed hypotheses and prepared the manuscript. AVV administered drugs and extracted brains. LMB selected the assays, developed analytical methods, and performed permeation assays and sample analyses. WAC provided training and assistance in sample collection. All authors participated in study design, and read and approved the final manuscript.

## Funding

National Institute of Mental Health (grant MH063266 to WAC); National Institute of Drug Abuse (DA09045 to FIC); Department of Defense (National Defense Science and Engineering Graduate Fellowship to AVV). The funders had no role in study design, data collection and analysis, decision to publish, or preparation of the manuscript.

## Supplementary Material

Additional file 1 Raw data for figures and tables as a Microsoft Excel spreadsheet. Readable by free software including LibreOffice and Google Docs. Click here for file
